# Dysfunctional Immune Regulation in Autoimmune Hepatitis: From Pathogenesis to Novel Therapies

**DOI:** 10.3389/fimmu.2021.746436

**Published:** 2021-09-28

**Authors:** Marta Vuerich, Na Wang, Ahmadreza Kalbasi, Jonathon J. Graham, Maria Serena Longhi

**Affiliations:** ^1^ Department of Anesthesia, Critical Care & Pain Medicine, Beth Israel Deaconess Medical Center, Harvard Medical School, Boston, MA, United States; ^2^ Department of Hematology, Shandong Provincial Hospital Affiliated to Shandong First Medical University, Jinan, China; ^3^ School of Medicine, Shandong University, Jinan, China

**Keywords:** autoimmune liver disease, Treg, Th17, ectonucleotidase, aryl hydrocarbon receptor

## Abstract

Autoimmune hepatitis (AIH) is a chronic inflammatory disorder characterized by hypergammaglobulinemia, presence of serum autoantibodies and histological features of interface hepatitis. AIH therapeutic management still relies on the administration of corticosteroids, azathioprine and other immunosuppressants like calcineurin inhibitors and mycophenolate mofetil. Withdrawal of immunosuppression often results in disease relapse, and, in some cases, therapy is ineffective or associated with serious side effects. Understanding the mechanisms underlying AIH pathogenesis is therefore of paramount importance to develop more effective and well tolerated agents capable of restoring immunotolerance to liver autoantigens. Imbalance between effector and regulatory cells permits liver damage perpetuation and progression in AIH. Impaired expression and regulation of CD39, an ectoenzyme key to immunotolerance maintenance, have been reported in Tregs and effector Th17-cells derived from AIH patients. Interference with these altered immunoregulatory pathways may open new therapeutic avenues that, in addition to limiting aberrant inflammatory responses, would also reconstitute immune homeostasis. In this review, we highlight the most recent findings in AIH immunopathogenesis and discuss how these could inform and direct the development of novel therapeutic tools.

## Introduction

Autoimmune hepatitis (AIH) was initially described as a severe form of fluctuating persistent hepatitis, associated with acneiform rashes, spider angiomas, amenorrhea and marked elevation of serum immunoglobulins (1). Subsequent studies showed that AIH occurs in individuals of all ages and, although present in both sexes, is prevalent in females (2).

AIH is diagnosed based on the presence of hypergammaglobulinemia, serum autoantibodies and histological features of interface hepatitis (3). While positivity for anti-nuclear antibody (ANA) and/or anti-smooth muscle antibody (SMA) identifies type-1 AIH (AIH-1), liver-kidney-microsomal-type-1 (LKM-1) antibodies, which specifically target the cytochrome P4502D6 (CYP2D6) liver enzyme (4), are the serological hallmarks of type-2 AIH (AIH-2) (5). Additional autoantibodies might aid in AIH diagnosis, especially in patients negative for ANA, SMA and LKM-1 autoantibodies. Antibodies to soluble liver antigen (SLA) have been reported in 58% of AIH patients where they are often associated with severe disease (6). Anti-liver cytosol type-1 (LC1) antibodies, which target formiminotransferase cyclodeaminase (FTCD), identify AIH-2 (7), and their titer positively correlates with disease activity (8). Presence of anti-neutrophil cytoplasmic antibody (ANCA), particularly atypical p-ANCA (pANNA), might also help in AIH diagnosis, in the absence of positivity for the above-mentioned autoantibodies (9).

Interface hepatitis is present at disease presentation in 84-98% of cases (10) and is characterized by a dense mononuclear cell infiltrate eroding the limiting plate and invading the liver parenchyma (11).

In 50% of cases, AIH manifests with an insidious onset that is often associated with lethargy, malaise, arthralgia, and myalgia; 30-40% of patients present with clinical features of acute hepatitis, whereas the remaining 10-20% of cases are incidentally discovered as having elevated transaminase levels on biochemical screening (12). AIH clinical manifestations may differ, depending on the geographical location and ethnicity of the affected patients. As an example, cirrhosis is more frequent in African Americans (56-85%) than Europeans (38%) (13, 14); and when considering subjects of non-European Caucasoid ethnicity, AIH has earlier onset in African, Arabian and Asian individuals, who also show lower response to immunosuppressive treatment (13, 14). Japanese patients are reported having a later onset of the disease that in most cases improves upon treatment with immunosuppressive drugs (15).

AIH can be associated with autoimmune disorders, which can be also found in first-degree relatives in 40% of cases (16).

The aim of AIH current treatment is to control inflammation. The management of the acute phase includes the administration of prednisone or prednisolone, which is gradually decreased to lower doses, based on the decline of transaminase levels. The addition of the anti-metabolite immunosuppressant azathioprine can be considered when the transaminase levels stop decreasing on steroid treatment alone or in the presence of steroid-related side effects (17). Relapse may occur in up to 40% of cases due to low compliance or when attempting treatment withdrawal. Additional drugs have been used as steroid-saving agents with the aim of reducing steroid-related side effects, these including cyclosporine and tacrolimus (18–20) and, in difficult-to-treat-cases, mycophenolate mofetil in association with prednisone or prednisolone (21, 22). None of these treatments, however, restores immunotolerance by boosting the impaired regulatory cell compartment.

Several studies have been conducted to identify the mechanisms involved in AIH pathogenesis. It has been proposed that genetically predisposed individuals upon exposure to certain environmental conditions (23) can develop cell-mediated immune responses against liver autoantigens. The derived inflammation, permitted by defective immune regulation, progressively results in fibrosis and cirrhosis with aberrant liver re-generation. Genetic predisposition to the disease is conferred by the presence of specific genes located within the human-leukocyte-antigen (HLA) region on the short arm of chromosome 6, especially those encoding allelic variants of DQB1 and DRB1 (24, 25).

Studies on AIH pathogenesis have been furthered by the generation of animal models, developed using different strategies. In this regard, immunization of C57BL/6 female mice with a pCMV plasmid containing the N-terminal region of mouse CTLA-4 and human CYP2D6 and FTCD resulted in transaminase elevations peaking 4 and 7 months after injection (26). Additional models, obtained upon infection of mice with adenovirus Ad5 expressing human CYP2D6, supported a role for viral infection as a possible mechanism leading to tolerance breakdown and consequent development of autoimmunity (27, 28). In an additional model by Hardtke-Wolenski et al, self-limiting adenoviral infection triggered immune responses against the human FTCD in mice of NOD background and resulted in chronic AIH that was characterized by portal and lobular fibrosis (29). Other AIH models have been obtained in mice with medullary thymic epithelial cell depletion due to a conditional deletion of Traf6 expression in murine thymic epithelial cells (30); and in PD-1^-/-^ mice upon neonatal thymectomy, which resulted in aberrant generation of follicular helper T-cells in the spleen (31). Overall, these studies indicate that experimental AIH may derive from different immunopathogenic routes that can be facilitated by viral infections and/or genetic predisposition, this recapitulating the human scenario.

From an immunological perspective, AIH liver damage is driven by overwhelming effector immune responses (32–35) that are not adequately controlled by suppressor/regulatory T-cells (Tregs) (36–39). Extracellular nucleotides and nucleosides are strong modulators of T-cell immunity (40). Mounting evidence links alterations of purinergic signaling with the immunological dysfunction present in autoimmune conditions (41). Both Treg and Th17-cells obtained from AIH patients express low levels and impaired activity of the immunoregulatory ectoenzyme CD39 (42, 43). We have recently reported that such dysfunction derives, at least in part, from alterations of aryl-hydrocarbon-receptor (AhR) signaling (44), suggesting that modulation of this pathway might be deployed to correct immunoregulatory defects while boosting Treg immunity in AIH.

Understanding the mechanisms underlying AIH immune dysregulation is of critical importance for developing more effective treatments. In this review, we will present and discuss the most recent experimental evidence of disrupted AhR/purinergic interactions as one of the prominent factors leading to immunotolerance breakdown in AIH. We will also review how these alterations might inform and direct towards novel therapeutic approaches that represent promising candidates for the treatment of AIH.

## Mechanisms of Liver Damage

The mechanisms underlying the liver attack in AIH are still unclear. The autoimmune reaction is believed to be initiated by the presentation of a liver autoantigen by antigen presenting cells (APCs) to Th0 lymphocytes that upon antigen recognition become activated. There is evidence that, in AIH, HLA-class-II molecules are expressed not only on professional APCs but also on hepatocytes (45), this favoring the amplification of cellular immune responses. Following activation, Th0 lymphocytes can differentiate into Th1, Th2 or Th17-cells, this depending on the cytokines in the milieu. All these subsets are present in the hepatic inflammatory infiltrates of AIH patients (35, 46). The cytokines released by each cell subset lead to a cascade of events culminating with the maturation of B-lymphocytes into plasma-cells and consequent production of autoantibodies, which are involved in antibody-mediated-cell-cytotoxicity (47). Derived immune reactions include activation of cytotoxic T-cells with subsequent release of IL-2 and IFNγ, activation of macrophages and, importantly, upregulation of HLA-class-I and class-II molecules by hepatocytes (45). Th17-cells are also involved in AIH liver damage, by perpetrating inflammation and through induction of pro-fibrotic events (35). If this cascade of events is not opposed by effective immune regulation, perpetration of immune attack occurs with consequent progression of tissue damage.

There is evidence that the frequency of liver autoantigen-specific T-cells present in the portal infiltrate is low (33, 48); these cells could orchestrate the liver damage by favoring the recruitment of non-antigen specific lymphocytes that, in turn, carry on hepatocyte damage by producing IFNγ and other cytotoxic factors.

Following the identification of CYP2D6 as the target autoantigen of LKM-1 autoantibodies in AIH-2 (4), several investigations have been conducted to identify CYP2D6 epitopes recognized by B and T-cells. By performing a conformational epitope mapping of the CYP2D6 molecule, Ma and colleagues identified CYP2D6_316-327_ as key target for autoantibodies (49). Subsequent studies performed by the same group showed that CD4 T-cell immunity in AIH-2 was polyclonal and involved multiple subsets of effectors with IFNγ, IL-4 and IL-10 producing lymphocytes targeting specific epitope regions within CYP2D6 (32). CD4-mediated immune responses were associated with liver damage, a finding also confirmed when analyzing CYP2D6-specific CD8 T-cell immunity (33). Future investigations should elucidate where autoantigen specific CD8 T-cell immune responses are initiated and perpetuated, given previous animal studies showing poor cytotoxic function and pro-inflammatory cytokine secretion in CD8 T-cells activated within the liver microenvironment (50); and additional studies showing that the spleen could modulate immune regulation as well as the intensity of hepatic inflammation (51, 52).

There is evidence that overwhelming effector cell immunity in AIH results from failure of immune regulatory mechanisms permitting the autoimmune damage to unfold and perpetuate.

Defects in regulatory cells have been studied extensively over the years, although the reasons leading to these impairments and the factors contributing to it remain still unclear.

## Treg Impairment in AIH: the Role of CD39

A wealth of studies has provided evidence that impairment of regulatory cells plays a permissive role in the initiation and progression of autoimmune tissue damage.

Among lymphocytes with immunoregulatory/suppressive properties, CD4^+^CD25^high^FOXP3^+^ Tregs have been those most extensively studied in the last decades. These cells play a key role in promoting and maintaining immunotolerance, by controlling effector immune responses. Tregs can be classified based on their developmental pathway. Generation of thymic Tregs (tTregs) is facilitated by intermediate affinity self-peptides/MHC interactions resulting in high intensity TCR signals (53). Peripheral Tregs (pTregs) can differentiate from T-cells in certain peripheral sites like the gut mucosa and acquire stable FOXP3 expression (54); whereas induced Tregs (iTregs) can be derived *in vitro* following Tconv exposure to suboptimal antigen stimulation in the presence of anti-inflammatory mediators (55, 56).

Tregs can suppress immune responses by different mechanisms that involve the downregulation of costimulatory molecules on APCs (57), release of cytokines like TGF-β (58), IL-10 (59) and IL-35 (60); activation of apoptosis (61), including the Galectin-9/T-cell-immunoglobulin-and-mucin-domain-3 (Tim-3) pathway (62); release of Granzymes and perforin (63); metabolic disruption *via* IL-2 deprivation from the environment (64); transfer of cyclic-adenosine-monophosphate (cAMP) to effector cells *via* gap junctions and subsequent upregulation of the inducible cAMP early repressor (65); or *via* CD39, an ectoenzyme that hydrolyzes pro-inflammatory ATP and ADP to ultimately generate immunosuppressive adenosine (40).

*In vivo* and *in vitro* studies have indicated that Treg impairment plays a key role in the pathogenesis of autoimmune diseases, permitting overwhelming effector cell immunity to perpetrate and perpetuate tissue damage (66–70).

Defects in suppressor lymphocytes/Tregs have been reported also in AIH and found to play an important role in permitting effectors like CD4 and CD8-cells to inflict hepatocyte damage (36–39, 71). Treg defects in AIH are multifactorial and include numerical impairment (37, 38, 72), functional defects (71, 73), high rate of immune exhaustion (74) as well as plasticity with tendency to acquire effector cell features, when exposed to or challenged with a proinflammatory stimulus (42). Subsequent investigations did not support these findings, likely as a result of differences in methodologies, patients’ demographics and clinical characteristics (75, 76).

Treg numerical impairment has been shown also in mice (77, 78). In a humanized mouse model of AIH, obtained upon injection of human CYP2D6/FTCD fusion protein into HLA-DR3^-^ or HLA-DR3^+^ transgenic NOD recipients, Yuksel and colleagues showed that defective Tregs were associated with heightened Th1-cell immunity with HLA-DR3^+^ mice undergoing the most severe form of the disease (78). In another study by Lapierre et al, adoptive transfer of *ex vivo* expanded CXCR3^+^ Tregs restored peripheral tolerance to FTCD and induced AIH remission (77).

**Figure 1:**
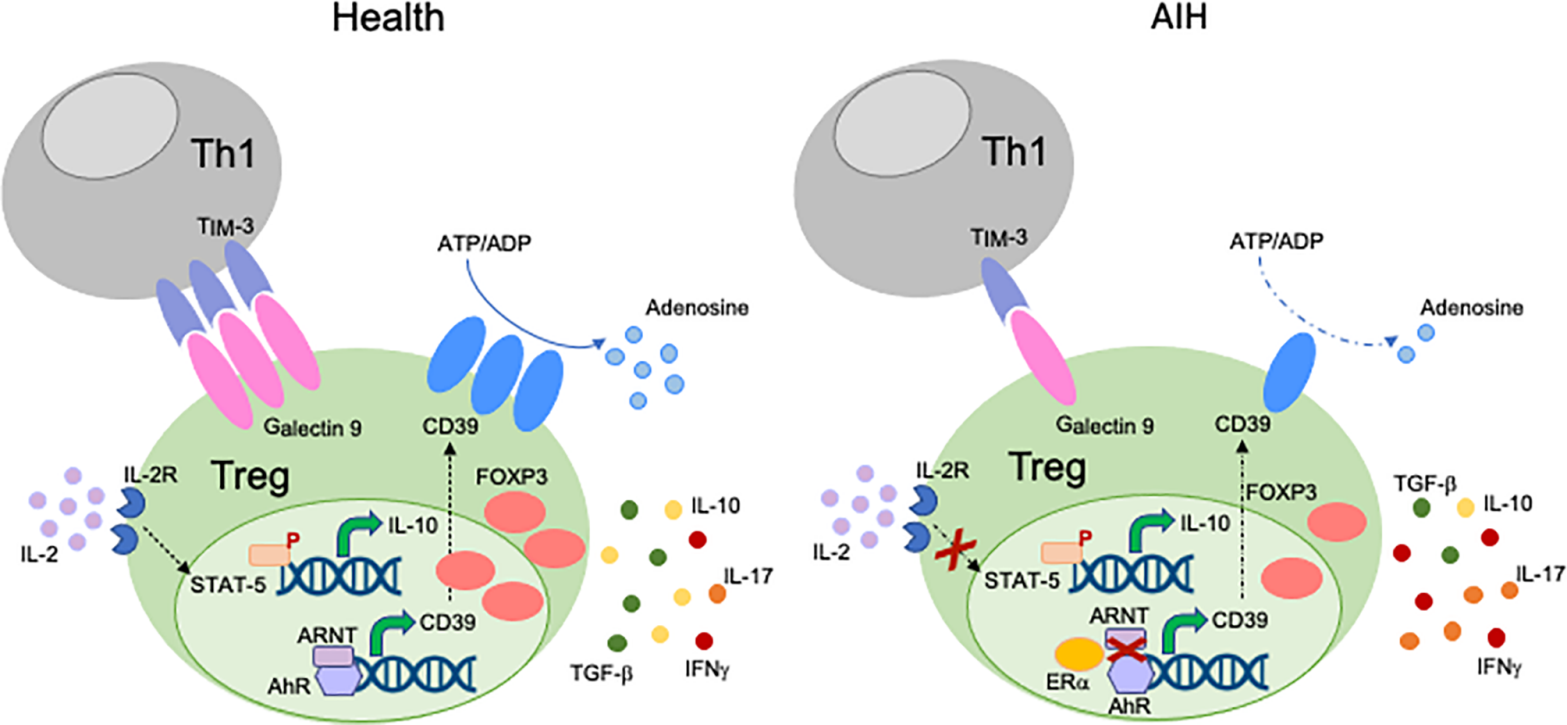
Mechanisms of Treg impairment in AIH. Different mechanisms account for Treg impairment in AIH. Low Galectin-9 in Tregs is associated with decreased TIM-3 levels on Th1-cells. This possibly results in reduced control of the effector potential of the Th1 subset through induction of apoptosis. AIH Tregs are also defective in their response to low dose IL-2, as reflected by impaired upregulation of pSTAT-5. AIH Tregs produce low amounts of IL-10 and TGF-β, while secreting effector cytokines like IFNγ and IL-17. This indicates that, in AIH, Tregs have high tendency to acquire effector cell features. AIH Tregs also display low levels and activity of CD39, an ectoenzyme that initiates a hydrolysis cascade culminating with the generation of immunosuppressive adenosine. In health, levels of CD39 are regulated by the aryl-hydrocarbon-receptor (AhR)/aryl-hydrocarbon-receptor-nuclear-translocator (ARNT) complex. In AIH, preferential AhR binding to estrogen-receptor-alpha (Erα), an AhR non-canonical partner results in less effective upregulation of CD39 by Tregs.

Different mechanisms might account for Treg impairment in AIH (**Figure 1**). We have shown that CD4^+^CD25^high^ Tregs from AIH patients display low levels of Galectin-9, contain higher frequencies of IFNγ^+^ and IL-17^+^-cells, while displaying lower proportions of FOXP3^+^, IL-10^+^ and TGF-β^+^-cells. This impairment is associated with low expression of Tim-3, the receptor of Galectin-9 on effector CD4-cells (39); this implicating that regulatory cell defects are linked with low responsiveness of T-effectors to Treg control. In a subsequent study we have reported that AIH Tregs display reduced ability to produce IL-10, this resulting in low responsiveness to low dose IL-2, as demonstrated by inability to upregulate the phosphor-signal-transducer-and-activator-of-transcription-5 (pSTAT5) (73).

In *de novo* AIH occurring after liver transplant Treg impairment was proposed to derive from high secretion of IL-12 and IL-6 by monocytes/macrophages that induced aberrant IFNγ production by Tregs impacting their function (79, 80). Blockade of TLR2 and TLR4 on monocytes resulted in reduction of IFNγ production by Tregs, further supporting the role of monocytes/macrophages in conferring Tregs proinflammatory features (79, 80).

In a mouse model of AIH, characterized by hepatocellular expression of a MHC-class II restricted immunodominant epitope of the lymphocyte choriomeningitis virus and by accumulation of CD4 T-cells specifically recognizing this epitope, Preti et al. proposed that liver damage was fostered by selective failure of peripherally induced autoreactive Tregs (81). Notably these autoreactive Tregs not only were reduced in frequency but also displayed heightened IL-17 production and reduced epigenetic demethylation (81), postulating a role for altered epigenetic regulation in Treg impairment in this model. In human AIH, *FOXP3* demethylation - a typical feature of *bona fide* Tregs - was retained in some studies (75, 79) and altered in others, where AIH derived Tregs were highly methylated (82); this indicates that further studies are needed to clearly establish the role of FOXP3 epigenetic regulation in AIH Tregs.

In subsequent studies, we have shown that Tregs obtained from the peripheral blood of AIH patients display low levels of CD39 ectoenzyme (42). In addition to displaying low CD39 levels, AIH Tregs are defective in their ectoenzymatic activity, this likely impacting their suppressive function due to lower adenosine generation (42). Impaired suppressive function of CD39^+^ Tregs is associated with increased tendency of these cells to upregulate CD127 and producing higher levels of IL-17 and IFNγ, when exposed to anti-CD3/CD28 (42).

CD39 can be regulated at genetic, transcriptional and post-transcriptional levels. Among the factors that regulate CD39 expression at the transcriptional level, the AhR signaling plays a pivotal role. AhR is a modulator of toxin responses that also regulates effector and Treg immunity (83). AhR is activated by endogenous ligands including products of heme catabolism like unconjugated bilirubin (UCB), tryptophan metabolites, 2-(1’ H-indole-3’-carbonyl)-thiazole-4-carboxylic acid methyl ester, dietary compounds like quercetin, or environmental pollutants, i.e., dioxin and benzo-*a*-pyrene. Upon binding to its ligands, AhR translocates to the cell nucleus where it forms a complex with the aryl-hydrocarbon-receptor-nuclear-translocator (ARNT), the AhR canonical partner, to regulate various genes, including cytochrome P450 enzymes, cytokines (IL-22, IL-17, IL-10), drug transporters (ABCB1, ABCC4) and CD39.

Notably, AhR activation has been demonstrated playing a role in the pathogenesis of other liver diseases, like hepatitis C, where the AhR-cytochrome P4501A1 pathway was found to favor lipid accumulation along with virus replication and assembly (84); and primary biliary cholangitis (PBC) where dioxin activated dendritic cells promoted differentiation of naïve CD4-cells, derived from PBC patients, into effector Th1 and Th17-cells (85). Importance of xenobiotics as triggers for PBC was also reported in earlier investigations (86, 87). Conversely, in the setting of experimental primary sclerosing cholangitis, administration of indole-3-carboxaldehyde could alleviate hepatic inflammation and fibrosis upon activation of the AhR-IL-22 axis (88). Further, kynurenine, a tryptophane metabolite that serves as AhR endogenous ligand, was decreased in the serum of children with AIH, when compared to controls (89).

Given the important links between AhR and CD39, we tested whether defective CD39 levels could derive from decreased AhR or ARNT expression. We found that AIH Tregs display AhR and ARNT levels comparable to Tregs derived from healthy controls; notably AIH Tregs express high levels of estrogen-receptor-alpha (Erα) (44), an AhR non-canonical partner. Further, in AIH Tregs, AhR preferentially binds to Erα rather than ARNT, this potentially resulting in less effective upregulation of CD39 upon exposure to ligands like UCB, L-kynurenine and quercetin (44). As increased Erα levels are present in patients under immunosuppression, we postulate that immunosuppressive drugs, while enabling control over inflammation, might also promote liver damage perpetuation by upregulating Erα and leading to a less effective AhR activation cascade. As silencing of Erα results in increased frequency of CD39^+^-cells among Tregs (44), strategies potentially interfering with AhR non-canonical binding might have a role in boosting CD39 levels, by restoring AhR signaling.

There is evidence that AhR transcriptional efficiency could be regulated by factors that impact the chromatin structure (90), suggesting that AhR can undergo epigenetic regulation. Previous studies have documented global hypomethylation of CD4 T-cells in systemic lupus erythematosus, and systemic sclerosis (90, 91); it remains to be established whether these mechanisms are operative also in CD4 T cell subsets in AIH, possibly impacting AhR function and its ability to regulate downstream genes like CD39.

## Aberrant CD39 Regulation in AIH Th17-Cells

Th17-cells are an effector subset that derives from CD4 lymphocytes upon exposure to IL-6 and TGF-β in mice and IL-6, IL-1β and TGF-β in humans (92). IL-23 plays an important role in the maintenance and stabilization of already differentiated Th17-cells (92). Transcription factors involved in the development of Th17-cells include RORγt, RORα and STAT-3 (92). Th17-cells can be regulated by additional factors, like AhR, which modulates CD39 and drug transporter levels (93, 94). Th17-cells have been involved in the pathogenesis of various autoimmune disorders including AIH (35).

**Figure 2:**
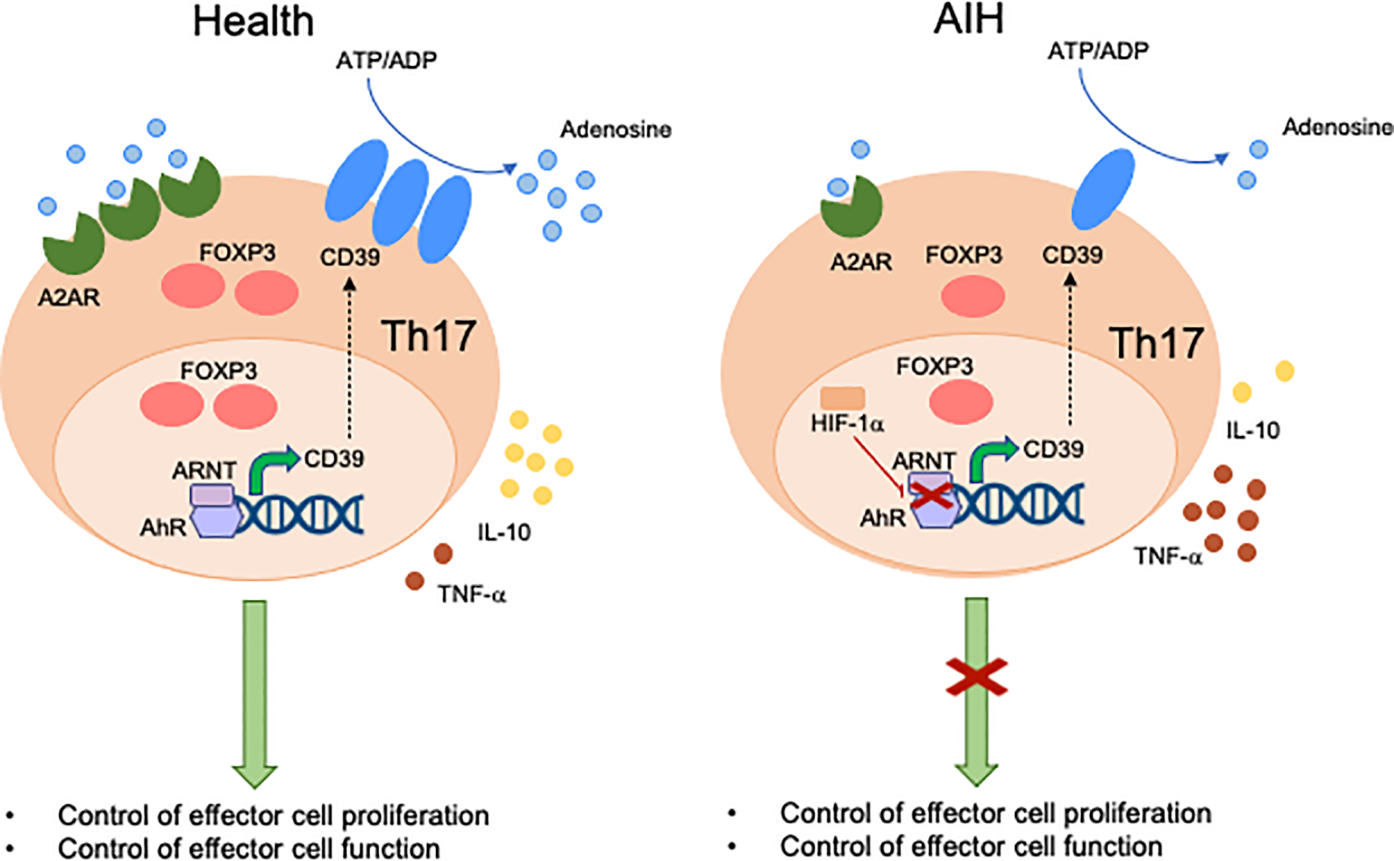
Mechanisms of aberrant Th17-cell immunity in AIH. Expression of CD39 by Th17-cells is associated with acquisition of regulatory properties. These include increased FOXP3 expression levels, IL-10 production and control of effector cell proliferation and function. In healthy subjects, levels of CD39 in Th17-cells are regulated by the aryl-hydrocarbon-receptor (AhR)/aryl-hydrocarbon-receptor-nuclear-translocator (ARNT) complex. In AIH, Th17-cells display decreased CD39 levels and activity. This is linked, at least in part, with high levels of hypoxia-inducible-factor-1alpha (HIF-1α) that inhibits AhR levels and signaling. Th17-cells from AIH patients also display reduced levels of A2A adenosine receptor, this implicating defective response of these cells to the immunosuppressive effects of adenosine.

Expression of CD39 by Th17-cells has been associated with attenuation of their pathogenic potential and acquisition of regulatory properties, as reflected by upregulation of FOXP3 and IL-10 production (93). Levels of CD39 in Th17-cells are regulated, at least in part, upon engagement of AhR by exogenous and endogenous ligands (93). Akin to Tregs, Th17-cells obtained from the peripheral blood of AIH patients express low CD39 levels and impaired ectoenzymatic activity (**Figure 2**). These defects are associated with reduced expression of A2A adenosine receptor, further confirming alterations in purinergic signaling mediators and, consequently, decreased response to adenosine. Due to lower generation of adenosine, AIH Th17-cells display impaired acquisition of regulatory properties that result in defective ability to control CD4^+^ CD25^–^cell proliferation and IL-17 production. As for Tregs, Th17-cells obtained from AIH patients cannot effectively upregulate CYP1A1 and CD39 when exposed to AhR ligands like UCB, L-kynurenine and quercetin, postulating alterations of AhR signaling as possible determinants of impaired CD39 levels. Differently from Tregs, however, no differences have been found in the expression of Erα. Instead, increase in the expression of hypoxia-inducible-factor-1alpha (HIF-1α), which is known to negatively regulate AhR expression (95) and signaling (94), is noted.

As discussed, the AhR signaling alterations noted in AIH impact both Treg and Th17 cell ability to upregulate CD39, this accounting for their impaired suppressive function (Tregs) and inability to acquire regulatory properties (Th17-cells). This indicates that defective immune regulation involves not only the regulatory but also the effector cell compartment. Strategies aimed at restoring alterations of AhR signaling both in Tregs and Th17-cells should be developed to guarantee a broader control over effector cell immunity in this autoimmune setting.

## New Immunotherapies

**Figure 3:**
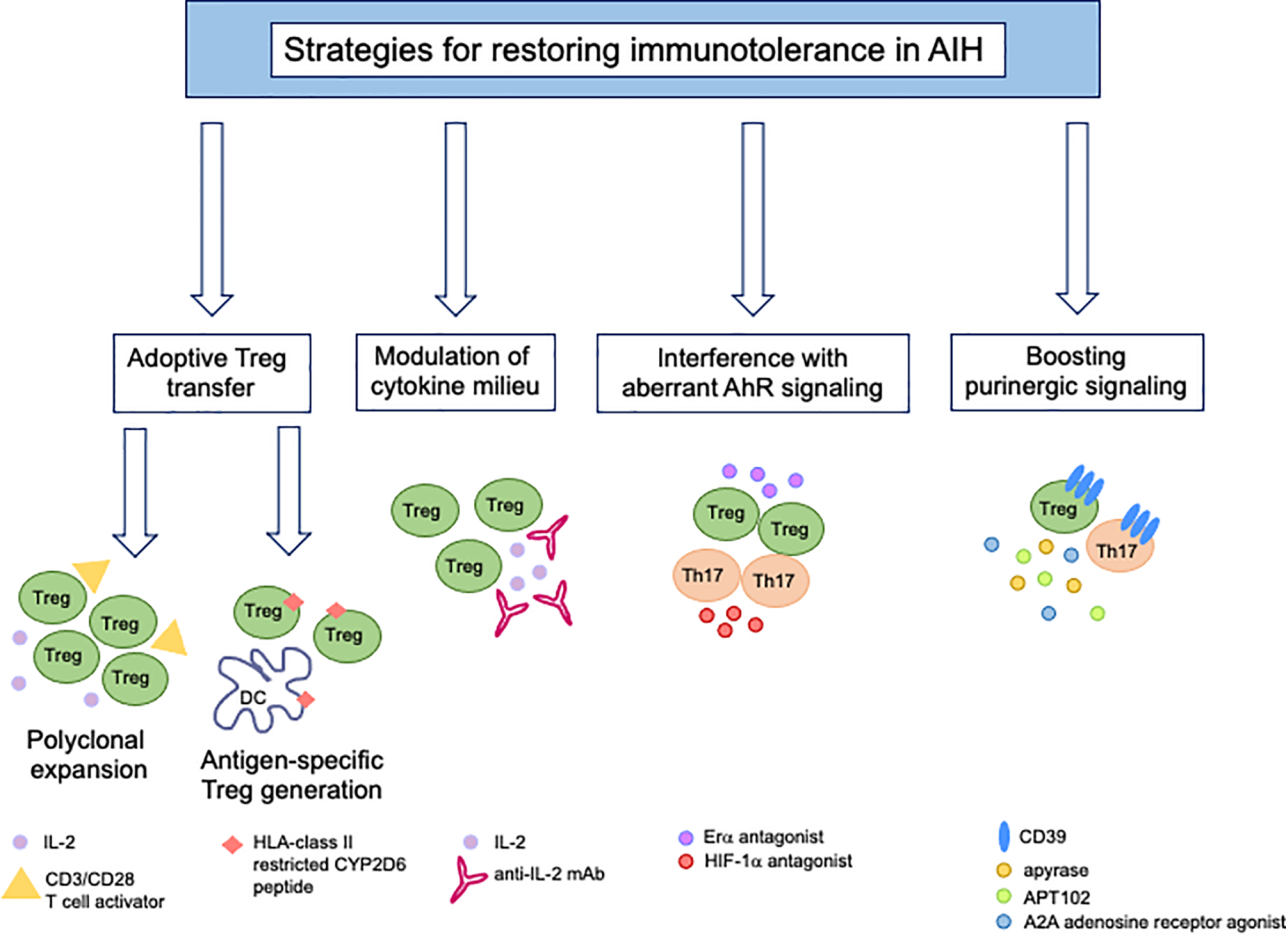
New immunotherapies for tolerance reconstitution in AIH. Strategies that might be considered as tools to re-establish immunotolerance in AIH include: adoptive transfer of Tregs, polyclonally expanded *in vitro* in the presence of high dose IL-2 and CD3/CD28 T-cell activator or generated under antigen-specific conditions upon co-culture with semi-mature dendritic cells; modulation of the cytokine milieu by administration of low dose IL-2 or monoclonal antibody/IL-2 complex; use of Erα or HIF-1α antagonists to interfere with aberrant AhR signaling in Tregs (Erα) and Th17-cells (HIF-1α); boosting of purinergic signaling through the use of exogenous apyrase, APT102, an ADPase that was found to enhance the beneficial effects of AhR activation *in vivo* and *in vitro*, and A2A adenosine receptor agonists to amplify the immunosuppressive properties of adenosine.

Given the multiple defects underlying immunotolerance breakdown in AIH, therapeutic strategies should be developed with the aim of restoring the pool of Tregs while dampening the effector potential of these cells (**Figure 3**). Reconstitution of the Treg pool could be achieved upon adoptive transfer. In previous studies we assessed Treg ability to expand under polyclonal conditions (96). Tregs expanded from the already existing regulatory cell pool express higher FOXP3 levels and suppress effector cells more effectively (96). This strategy also resulted in *de novo* generation of Tregs from CD4^+^CD25^-^ effector lymphocytes, when using healthy controls and AIH-derived cells (96). In line with this strategy, inhibition of IL-17 was found to favor the differentiation of newly generated Tregs from CD4^+^CD25^-^ effectors (97), supporting the postulate that immune regulation could be enhanced even further when effector cells are conditioned to become suppressive. Exposure of CD127^+^CD25^+^ activated cells to TGF-β and IL-2 boosts IL-10 production and suppressive function of these cells (98). As CD39 is strictly linked with Treg ability to suppress, adoptive transfer of CD39^high^ Tregs (99) would be preferable; however, since this subset is substantially impaired in AIH patients (42), strategies that enable boosting CD39 in Tregs, prior to their transfer, should be implemented. A study by Oo and colleagues showed that polyclonal Tregs, isolated from leukapheresis products, labelled with indium tropolonate and re-infused in AIH patients, homed to the liver where they were detected for up to 72 hours (100). In a murine model of AIH in the context of autoimmune-polyendocrine-syndrome-type-1, a multiorgan autoimmune condition caused by mutations of the autoimmune regulator (AIRE) gene, adoptive transfer of polyspecific Tregs was effective in treating AIH (101).

In the context of AIH-2 where the autoantigen and the CD4 and CD8 T-cell epitopes within it are known, adoptive transfer of autoantigen-specific Tregs would enable achieving a tailored and effective form of treatment to re-establish immunotolerance. In this regard, we could generate Tregs specific for HLA-DR3 and HLA-DR7 restricted CYP2D6 epitopes upon co-culture with semi-mature DCs that enable antigen presentation and promote immunotolerance induction (102). CYP2D6-Tregs suppress the proliferation and pro-inflammatory cytokine production by CD4 effectors of the same antigen specificity (102). As CYP2D6-specific Tregs showed the tendency to lose their suppressor properties when exposed to proinflammatory stimuli, treatment with retinoic acid could stabilize their phenotype and functional properties even in the presence of an inflammatory milieu (103). Effort has been made in subsequent studies to control autoantigen-specific effectors upon *in vivo* injections of peptide-major histocompatibility class-II (pMHC-II) nanomedicines with specificity for the immunodominant epitope CYP2D6_398-412_ in a mouse model of AIH, obtained by infecting NOD mice with replication defective adenovirus encoding for human FTCD (104). This approach resulted in the expansion of Tr1-cells (104). Preclinical studies have provided evidence that antigen-specific Tregs deliver targeted and superior immunosuppression, when compared to other approaches, including polyclonal Tregs (105). Since generation of antigen-specific Tregs is, however, hampered by their limited ability to expand, recent studies have shown that these cells could be also obtained from polyclonal Tregs through transduction of Treg T-cell receptor or chimeric antigen receptor (CAR) (105); or, through strategies based on the CRISPR Cas9 system where an endogenous TCR can be replaced by a recombinant one (105).

In addition to cell-based therapies, the implementation of which may be limited by substantial costs, Tregs could be expanded by modifying the cytokine environment. A study by Diestelhorst et al. proposed that the marked decrease in AIH Tregs during immunosuppressive therapy might derive from decreased levels of IL-2, a cytokine key for Treg survival (106). Exposure of PBMCs from patients with autoimmune liver diseases, including AIH, to very-low-dose Proleukin, used at less than 5 IU/ml, resulted in phosphorylation of STAT-5 and increased levels of CTLA-4 and FOXP3 in the Treg subset (107). Administration of low dose IL-2 would enable *in vivo* cell reconstitution, even when the autoantigen is unknown. In a study on two AIH patients with persistent disease activity, administration of low dose IL-2 resulted in Treg expansion in both. This was evident on day 9 but returned to baseline levels on day 28 (108). Clinical response was observed in one case, suggesting that the effects of IL-2-induced Treg expansion is transient and the clinical benefit limited, at least in the small cohort studied. Additional efforts should be made to optimize and successfully implement this approach.

To prolong the half-life of IL-2 in the bloodstream, mutants have been developed to promote Treg expansion *in vivo*. Another strategy has been attempted and consists of the administration of monoclonal antibody/IL-2 complex to expand Tregs in a mouse model of colitis (109) and AIH (110). A new complex of IL-2 and anti-IL-2 monoclonal antibodies was found to stimulate Treg expansion among human T-cells *ex vivo* and in rhesus macaques *in vivo* (111).

Transient increase in the proportion of splenic Tregs was achieved in a mouse model of AIH, resulting from xenoimmunization of C57BL/6 mice with DNA coding for human liver autoantigens, after treatment with low-dose anti-CD3 antibody (112). No differences in the proportion of splenic and liver derived Tregs were noted in xenoimmunized mice subjected to anti-CD20 antibody treatment, this approach inducing AIH remission by decreasing antigen presentation (through B-cells) and help to T lymphocytes (113). Whether belimumab, an inhibitor of serum B-cell activating factor that has been recently proposed as a promising treatment option for patients with refractory AIH and advanced liver-related fibrosis, induces disease remission having also a beneficial impact on Treg frequencies, is unknow (114).

As immunoregulatory defects in AIH involve both Tregs and effector Th17-cells, future approaches targeting both cell types should be considered. Our research has shown impaired CD39 levels in both Tregs and Th17-cells obtained from the peripheral blood of AIH patients. Reduced CD39 expression is present in AIH patients during active disease and remission, suggesting an intrinsic defect of this ectoenzyme in AIH Treg and Th17 lymphocytes (42, 43). This defect derives, at least in part, from aberrant increase in the levels of Erα, an alternative AhR partner, which is upregulated in AIH Tregs; and from high expression of HIF-1α, an AhR inhibitor, which is upregulated in AIH Th17-cells. In this regard, strategies inhibiting Erα and HIF-1α may help reconstituting the altered AhR signaling in AIH, and along with this, CD39 and immunotolerance.

Alternatively, interventions that boost CD39 levels might represent an additional option to bypass the altered AhR signaling/pathway and restore immunotolerance by enhancing the levels and/or activity of this ectoenzyme.

These approaches could be based on exogenous apyrase that has ectoenzymatic activity comparable to CD39; and APT102, the extracellular domain with improved ADPase activity of human nucleoside-triphosphate-diphosphohydrolase-3, a member of the CD39 family (115). APT102 was found beneficial in enhancing the immunoregulatory properties of the AhR ligand UCB in an experimental model of colitis in mice and in promoting increased levels of immunoregulatory molecules on Tregs and Tr1-cells (115). Notably, there were no safety or toxicity concerns when APT102 treatment was protracted after resolution of colitis *in vivo* (115). Additional studies reported on the safety profile of this molecule in the vascular setting (116, 117). However, since CD39 also plays an important role in tumor development and progression (99, 118–121) and inhibition of effector immune responses against pathogens (99, 122, 123), the safety of long-term treatments aimed at restoring the levels of this ectoenzyme in the context of autoimmune diseases should be considered and carefully evaluated. A2A receptor agonists that enhance the adenosinergic signal might also have a role in restoring immunotolerance by reconstituting the purinergic milieu (41).

Strategies targeting CD39 antisense RNA, a long noncoding RNA regulating CD39 at both mRNA and protein levels, should be taken into consideration. Silencing of CD39 antisense RNA has been effective in containing disease activity in an experimental mouse model of colitis in humanized *NOD/scid/gamma* mice, reconstituted with human CD4-cells (124).

## Concluding Remarks

This review has discussed the mechanisms mediating liver damage perpetuation and progression in AIH with a specific focus on the imbalance between Tregs and Th17-cells.

Numerous pathways might be involved in AIH disordered immunity. We have discussed how defects in CD39 are linked with aberrant AhR signaling in AIH-derived Tregs and Th17-cells. Standard treatment of AIH is still based on corticosteroids and azathioprine, immunosuppressive drugs that currently enable control of inflammation without, however, restoring immunotolerance and preventing progression to end-stage liver disease. Strategies targeting factors that interfere with AhR canonical pathway or directly boosting CD39 expression and activity might represent new therapeutic avenues in the treatment of AIH.

## Author Contributions

MV, NW, and AK wrote the manuscript. JG performed literature search. ML reviewed and edited the manuscript. All authors contributed to the article and approved the submitted version.

## Funding

This work has been supported by the National Institutes of Health (R01 DK108894 and R01 DK124408 to ML) and the Department of Anesthesia Seed Grant Award (to ML).

## Conflict of Interest

The authors declare that the research was conducted in the absence of any commercial or financial relationships that could be construed as a potential conflict of interest.

## Publisher’s Note

All claims expressed in this article are solely those of the authors and do not necessarily represent those of their affiliated organizations, or those of the publisher, the editors and the reviewers. Any product that may be evaluated in this article, or claim that may be made by its manufacturer, is not guaranteed or endorsed by the publisher.
